# Supplementary material for: Dual-comb photoacoustic spectroscopy
Source: Nat Commun. 2020 Jun 19;11:3152. doi: 10.1038/s41467-020-16917-y (PMC7305174; doi:10.1038/s41467-020-16917-y)
Supplement: Supplementary file 1 — Supplementary Information [file 41467_2020_16917_MOESM1_ESM.pdf]

Supplementary Information for:

Dual-comb photoacoustic spectroscopy

Jacob T. Friedlein, Esther Baumann, Kimberly A. Briggman, Gabriel M. Colacion, Fabrizio R. Giorgetta, Aaron M. Goldfain, Daniel Herman, Eli V. Hoenig, Jeeseong Hwang, Nathan R. Newbury, Edgar F. Perez, Christopher S. Yung, Ian Coddington, Kevin C. Cossel*

Author for correspondence: kevin.cossel@nist.gov

Supplementary Note 1: Comparison between dual-comb photoacoustic spectroscopy and conventional photoacoustic spectroscopy

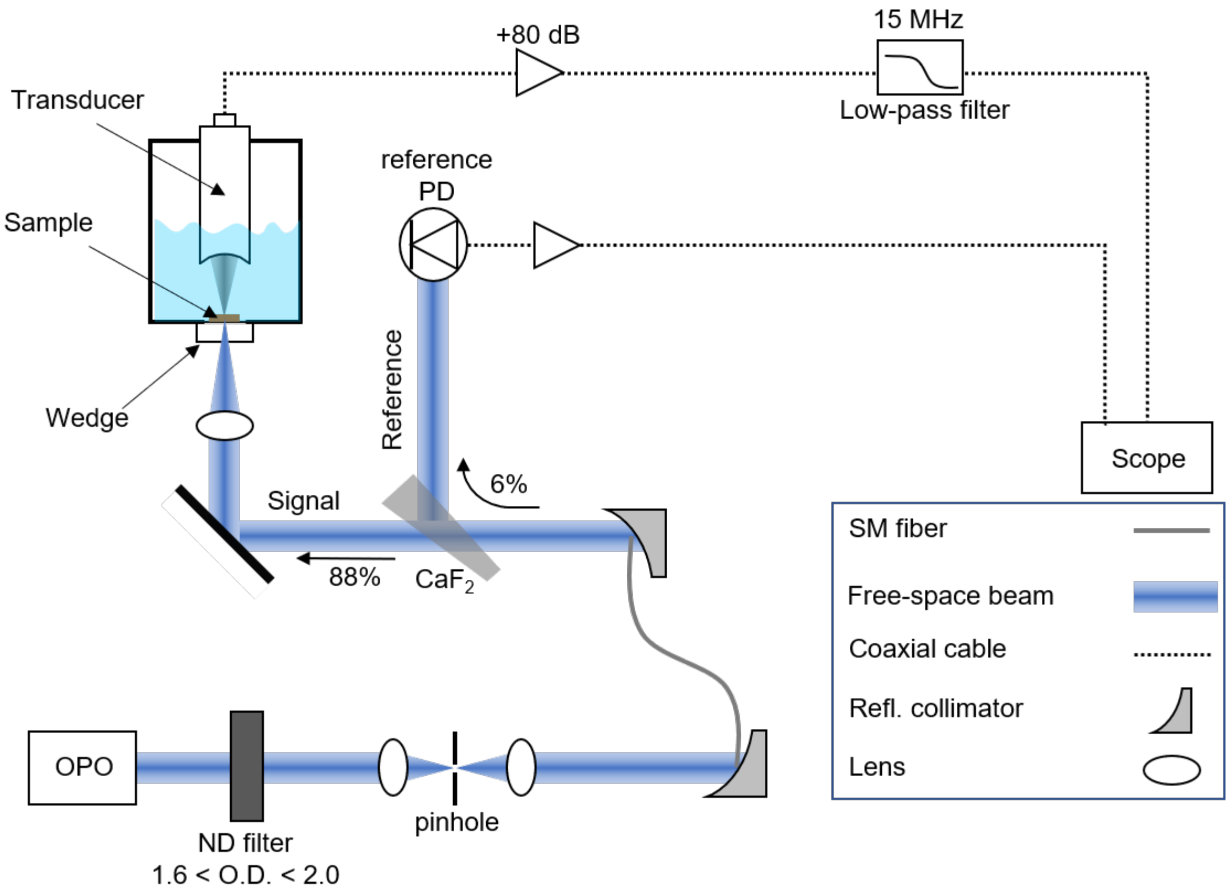

Supplementary Figure 1. Conventional photoacoustic spectroscopy comparison setup with single-wavelength, pulsed light source. This figure shows how the conventional photoacoustic light source was implemented in our measurement setup for comparison with dual-comb photoacoustic spectroscopy. OPO: optical parametric oscillator, ND: neutral density, PD: photodetector

As discussed in the main text, the current dual-comb photoacoustic spectroscopy (DCPAS) system is not optimized for SNR and was focused rather on demonstrating the basic approach. Nevertheless, it is important to consider the DCPAS SNR compared to conventional photoacoustic spectroscopy (PAS) measurements. It is challenging to conduct such a comparison based only on the available PAS literature because of the large number of parameters in any PAS experiment. Therefore, we have made as direct a comparison as possible by replacing the dual-comb source with a conventional PAS laser source. The modified experimental setup is shown in Supplementary Figure 1, which can be compared to Figure 2 of the manuscript. We first compare the SNR of the time-domain signals, acquired with the two systems. We then discuss spectral-domain SNR and how SNR scales with laser parameters for each system.

The PAS light source is a tripled YAG-pumped optical parametric oscillator (OPO) that produces 4-ns-duration pulses at a repetition rate of 10 Hz. The output of the OPO is coupled into a single mode fiber, collimated and then focused onto the sample. The collimator, focusing lens and sample are the same used in the DCPAS measurement. Pulse-to-pulse power fluctuations from the OPO are corrected by normalizing the PA signal from each pulse by the pulse energy measured on the reference photodiode (see

Supplementary Figure 1). We refer to this system as the OPO-PAS system. To obtain PA spectra, the pulsed OPO wavelength can be scanned and the PA response measured at each wavelength.

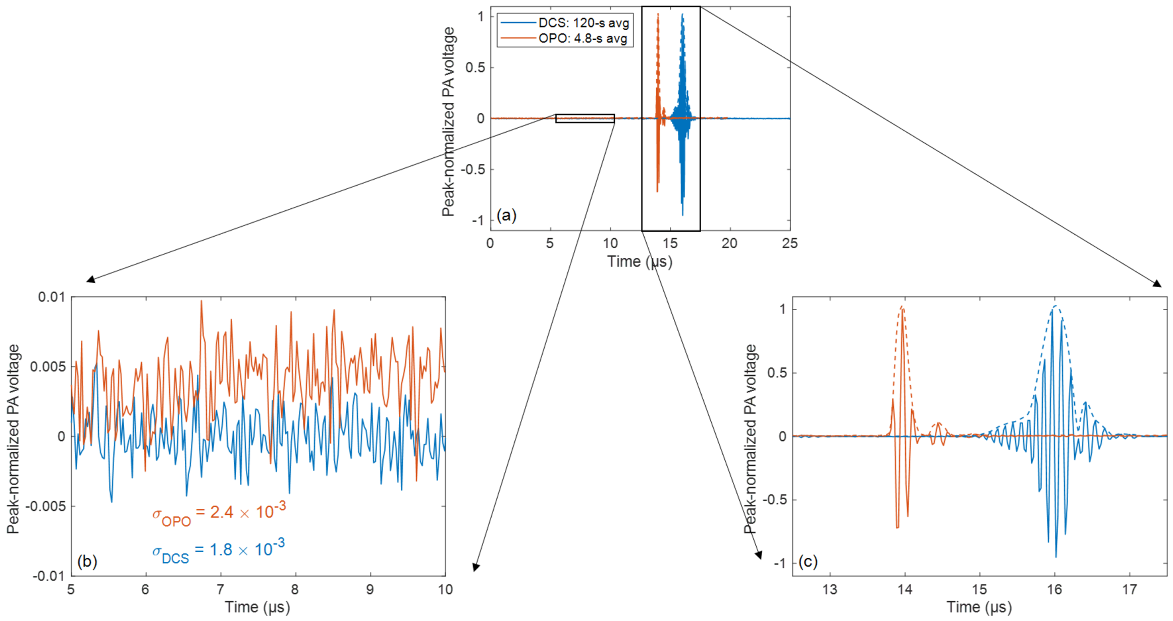

Supplementary Figure 2. Comparison of dual-comb and conventional photoacoustic spectroscopy. This figure shows the time-domain signals of VACNTs measured with DCPAS (blue) and OPO-PAS (PAS with OPO illumination) (red). The σ values listed in (b) are the standard deviations of the signals in the 5 μs to 10 μs window shown. Because the peak signals are normalized to unity, the time-domain peak SNR is simply the inverse of σ. For visual clarity, the DCPAS data in this figure are delayed by 2 μs to avoid overlapping with the OPO-PAS data. Dashed lines in (c) indicate the analytical envelope of the corresponding signal.

Supplementary Figure 2 shows the time-domain signals for DCPAS and OPO-PAS for the VACNT sample. The OPO-PAS signal is acquired at $\nu_{\mathrm{optical}} = 167$ THz and with a pulse energy of 20 nJ per pulse incident on the sample. The DCPAS signal is acquired for the full optical spectrum (165 THz to 181 THz) and an average power of approximately 25 mW incident on the sample. The signals are normalized to a peak height of 1, allowing facile comparison of the peak-normalized noise levels. The DCPAS signal is averaged over 120 s and the OPO-PAS signal is averaged over 4.8 s or 48 pulses. This difference in averaging time reflects the additional time that would be needed for the OPO-PAS to scan across the full spectrum acquired by the DCPAS, which measures all wavelengths simultaneously. Specifically, we assume a spectral resolution of 500 GHz over an approximately 12.5 THz spectral bandwidth of DCPAS illumination, corresponding to 25 spectral points. The required acquisition time for a full spectrum by the OPO-PAS would then be $25 \times 4.8\ \mathrm{s} = 120\ \mathrm{s}$ to match the DCPAS acquisition time. (This analysis assumes zero measurement deadtime associated with wavelength tuning the OPO, which is not necessarily realistic). In both cases, the noise is dominated by the transducer noise. As shown in Supplementary Figure 2, the peak time-domain SNR for the DCPAS is $SNR_{\mathrm{DCS}} = 1/\sigma_{\mathrm{DCS}} = 560$ and the peak time-domain SNR of the OPO-PAS is $SNR_{\mathrm{OPO}} = 1/\sigma_{\mathrm{OPO}} = 420$.

This same peak time-domain SNR is used in the main text in Figure 5 to investigate the scaling of the DCPAS SNR with power and integration time. In cases with very low SNR, for example for PDMS or paraffin at low comb powers or short integration times, the SNR can approach unity. In that case, the simple analysis above can lead to a bias because of the significant contribution of noise at the peak of the

interferogram. To avoid this bias, we apply a matched filter based on the interferogram from a high-SNR measurement of the same sample to extract the peak interferogram voltage.

To further compare the spectroscopic performance of OPO-PAS with DCPAS, the time domain SNR can be translated to spectral SNR. For DCPAS, Fourier transformation of a time-domain interferogram yields a spectrum, where the PA signal level at an optical frequency ν is simply the FFT amplitude at ν. To find the spectral SNR, we acquire a series of time-domain interferograms from which we calculate a series of spectra. The spectral SNR is the mean of the amplitudes at ν from the series of spectra, divided by the standard deviation of the amplitudes at ν. For OPO-PAS, the PA response at a given optical frequency ν is defined as the area under the envelope of the time-domain signal with OPO illumination at ν. The spectral SNR at optical frequency, ν, is then calculated in a similar manner from a series of measurements. In other words, the spectral SNR is the mean of the series of measurements divided by their standard deviation. For DCPAS, we find a peak spectral SNR of 285 at 174 THz with an excitation power of 25 mW and 120 seconds of averaging. For OPO-PAS, we find a peak spectral SNR of 420 at $\nu = 167$ THz with a pulse energy of 20 nJ and 4.8 seconds of averaging. Therefore, for equal averaging time per spectral point, the OPO-PAS spectral SNR and DCPAS spectral SNR were similar, although the OPO-PAS was better by a factor of $420/285 = 1.5$.

However, we would expect higher SNR with increased OPO power. The OPO output power was attenuated prior to coupling to avoid damaging the single mode fiber tip. As a result, the total pulse power was about 20 nJ, as given above. Based on other reports of fiber-coupled optical-resolution photoacoustic microscopy systems[1], pulse energies up to 1 µJ might be possible before damaging the single-mode fiber (sample damage might also limit the pulse energy for the tightly confined <10 µm optical focus). Assuming no saturation of the sample response, this higher power pulse would increase the OPO-PAS SNR by a factor of $1\ \mu\text{J}/20\ \text{nJ} = 50\ \times$. A similar SNR increase is possible for the DCPAS system if the frequency comb repetition frequency were better matched to the desired resolution. As noted in the manuscript, a 500 GHz frequency comb would increase the interferogram duty cycle by a factor of $500\ \text{GHz}/160\ \text{MHz} = 3000\ \times$, leading to an increase of the SNR by a factor of $\sqrt{3000} \approx 55\ \times$ for the same fixed averaging time. Again, we find that an optimized DCPAS would have a similar SNR to that obtained with an optimized, fiber-coupled OPO-PAS system.

There are additional important comparisons that extend beyond the scope of this work including sample response times, axial resolution, etc. For example, we found differences in the system-versus-system comparison due to sample geometry. We made a comparison between DCPAS and OPO-PAS for the PDMS sample similar to the comparison for VACNTs described above. We found that the PDMS sample yielded a DCPAS spectral SNR that is 3× lower than the spectral SNR obtained with OPO-PAS system. We believe that the DCPAS SNR is degraded for these measurements because of acoustic pulse broadening and/or destructive interference between acoustic signals generated at the top and bottom of the polymer film. These signals overlap for the longer DCPAS interferogram, but they are separately resolved for the narrower signal from the OPO-PAS system. Further experiments will be needed to understand the issues related to possible broadening or interference.

Supplementary Note 2: Transducer responsivity

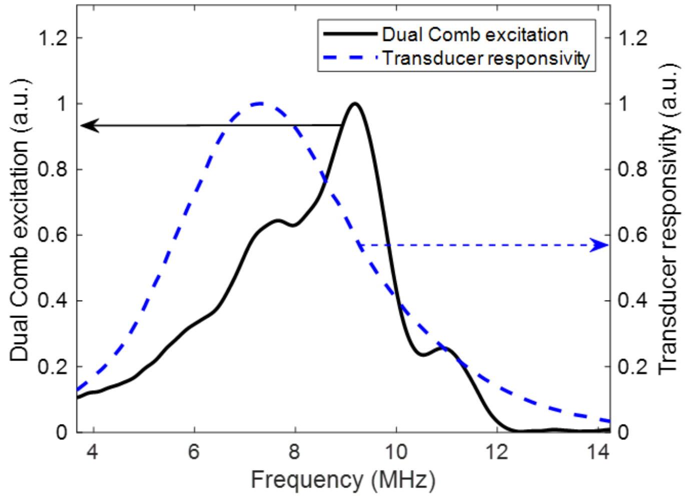

Supplementary Figure 3. Transducer responsivity and multiheterodyne illumination signal. Shown here is the transducer responsivity (blue dotted line) compared to the multiheterodyne frequencies generated by the dual-comb optical excitation (black solid line). Note that the agreement is not fortuitous. Rather, the relative repetition and offset frequencies of the two frequency combs are selected so that the multiheterodyne spectrum is matched to the transducer response. A transducer with a different bandwidth or center frequency could be accommodated by adjusting the frequency comb parameters. For instance, if the transducer spectrum narrows, one can similarly narrow the multiheterodyne spectral bandwidth by reducing the difference in repetition frequencies, assuming the two combs are sufficiently phase coherent.

Supplementary Note 3: Sample dimensions

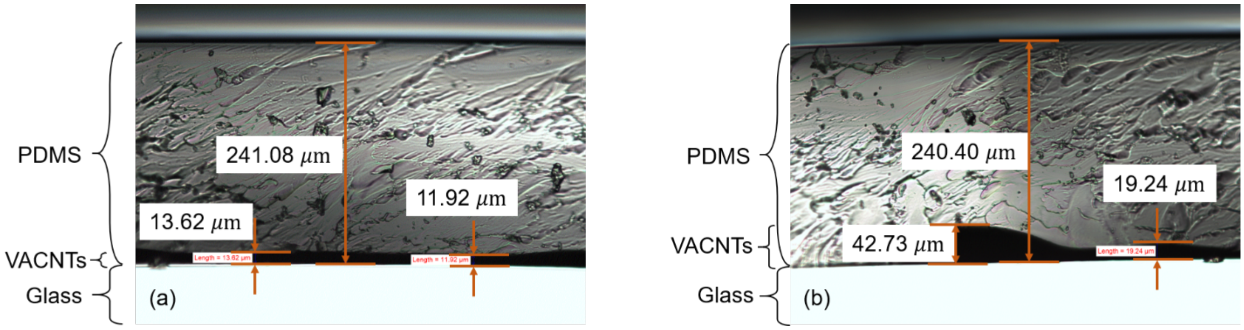

Supplementary Figure 4. Sample thickness. Shown here is a cross-sectional microscope image of PDMS/VACNT sample after slicing through the middle of the sample with a razor blade. (a) Near the center of the VACNT film. (b) Near the left edge of the VACNT film.

Using a razor blade, we sliced the sample with VACNTs embedded in PMDS. From this slice, we obtained a cross-sectional microscope image. Supplementary Figure 4 shows that the PDMS sample is ≈ 240 μm thick and that the VACNT layer thickness is ≈ 15 μm thick near the center and ≈ 40 μm thick near the edges. We measured the total absorption of the VACNTs in PDMS and found that, near the edge of the sample ≈ 5% of the incident light was transmitted through the sample and near the center of the sample ≈ 15% of the light was transmitted. For the paraffin sample, we measured its thickness using contact profilometry after preparing a test sample in the same way as the sample used for our PAS measurements. We found the paraffin thickness to be ≈ 120 μm. Note that the scratches and

inhomogeneities on the face of the PDMS cross-section were induced by cutting the sample with the razor blade to show the cross section and not present in the as cast, bulk PDMS film. Therefore, these inhomogeneities did not affect our measurements.

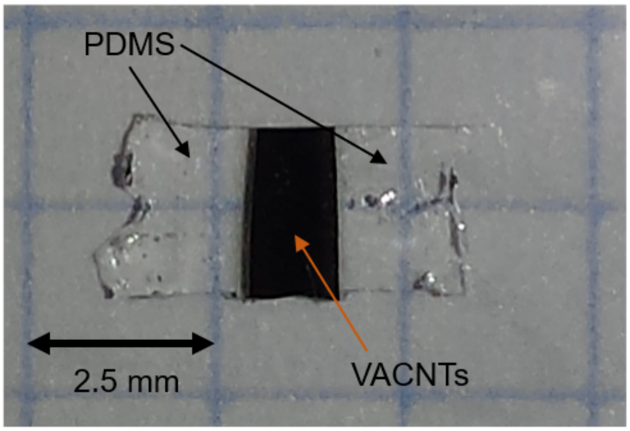

Supplementary Figure 5. Sample lateral dimensions. This figure shows a top-down image of the VACNTs and PDMS. For measurements of VACNTs, the illumination was contained entirely in the region covered by VACNTs. For measurements of PDMS, the illumination was contained entirely in the region with no VACNTs.

For spectrophotometer measurements, the PDMS sample was from the same PDMS used for DCPAS in a region with no VACNTs, although the spectrophotometer illuminated a different portion of the sample than the dual-comb excitation. The paraffin sample used for DCPAS could not be used for spectrophotometer measurements because it displayed too much optical scattering for transmission measurements. We reduced the optical scattering by placing the paraffin between two glass microscope slides, heating the glass to 70 C, and applying pressure. Spectrophotometer measurements of this sample were made after allowing it to cool to room temperature.

Supplementary References

1. Hajireza, P., Forbrich, A. & Zemp, R. *In-vivo* functional optical-resolution photoacoustic microscopy with stimulated Raman scattering fiber-laser source. *Biomed. Opt. Express* **5**, 539-546 (2014).
